# Research on the Health Assessment Method of the Safety Retaining Wall in a Dump Based on UAV Point-Cloud Data

**DOI:** 10.3390/s23125686

**Published:** 2023-06-18

**Authors:** Yachun Mao, Xin Zhang, Wang Cao, Shuo Fan, Hui Wang, Zhexi Yang, Bo Ding, Yu Bai

**Affiliations:** School of Resources & Civil Engineering, Northeastern University, Shenyang 110819, China; maoyachun@mail.neu.edu.cn (Y.M.); 2010418@stu.neu.edu.cn (W.C.); 2000971@stu.neu.edu.cn (S.F.); 2000981@stu.neu.edu.cn (H.W.); 2100971@stu.neu.edu.cn (Z.Y.); 2201042@stu.neu.edu.cn (B.D.); 2210436@stu.neu.edu.cn (Y.B.)

**Keywords:** UAV point-cloud data, safety retaining wall, elevation gradient filtering, ordered criss-crossed scanning algorithm, surface reconstruction, health assessment

## Abstract

The safety retaining wall is a critical infrastructure in ensuring the safety of both rock removal vehicles and personnel. However, factors such as precipitation infiltration, tire impact from rock removal vehicles, and rolling rocks can cause local damage to the safety retaining wall of the dump, rendering it ineffective in preventing rock removal vehicles from rolling down and posing a huge safety hazard. To address these issues, this study proposed a safety retaining wall health assessment method based on modeling and analysis of UAV point-cloud data of the safety retaining wall of a dump, which enables hazard warning for the safety retaining wall. The point-cloud data used in this study were obtained from the Qidashan Iron Mine Dump in Anshan City, Liaoning Province, China. Firstly, the point-cloud data of the dump platform and slope were extracted separately using elevation gradient filtering. Then, the point-cloud data of the unloading rock boundary was obtained via the ordered crisscrossed scanning algorithm. Subsequently, the point-cloud data of the safety retaining wall were extracted using the range constraint algorithm, and surface reconstruction was conducted to construct the Mesh model. The safety retaining wall mesh model was isometrically profiled to extract cross-sectional feature information and to compare the standard parameters of the safety retaining wall. Finally, the health assessment of the safety retaining wall was carried out. This innovative method allows for unmanned and rapid inspection of all areas of the safety retaining wall, ensuring the safety of rock removal vehicles and personnel.

## 1. Introduction

The emergence of the “Intelligent Mine” [1] concept has led to the development of highly intelligent and unmanned mines. The foundation of an Intelligent Mine is based on mine digitalization [2] and informatization [3], which allows for the active perception, automatic analysis, and rapid processing of mine production and safety data. The ultimate goal of an Intelligent Mine is to construct a safe, efficient, unmanned, and environmentally friendly mine [4,5].

As a critical infrastructure within an open pit production system, a dump serves as a centralized discharge site for mine stripping and excavation waste. However, it is also a large, artificial pile of loose material that can pose significant safety hazards, disrupting mine production, causing economic losses, and posing a risk to workers’ lives in the event of a safety accident. Landslides, caused by precipitation infiltration, earthquakes, and other triggers of instability, are common safety incidents in dumps. For example, the Yelbashinskii dump at the Kolyvan anthracite open pit in Novosibirsk, Russia, experienced landslides due to earthquakes [6], while a dump in Wuhai, Inner Mongolia, China, suffered multiple slope failures and landslides following heavy rainfall [7]. In response to these hazards, many scholars from both home and abroad have conducted extensive research and analysis on dump safety. For instance, Nguyen Phu Minh Vuong et al. [8] employed numerical modeling to analyze slope stability in the Janina mine waste dump located in Libiąż, Poland, under the impact of rainfall. Similarly, Tian Ya et al. [9] modified the meso-mechanical parameters obtained through discrete element numerical simulation using slope radar monitoring data to determine the stability factor of safety for different heights of steps in the dump. Lai Shan Chang et al. [10] analyzed the geological conditions and failure patterns of constructing a 100 m high dump on the QIANYU abandoned tailing pond to predict potential disasters to nearby villages and industrial buildings and determined corresponding preventive measures. Furthermore, dumps are not only prone to geological hazards, but also to safety accidents during dump operations. Earthwork operations, such as soil and rock excavation, transportation, and unloading, are the main tasks in open-pit mining, and rock removal vehicles soil unloading is one of the key and most dangerous operations among them. The rock removal vehicles back up to the edge of the dump platform in the dump and unload soil and rock materials along the slope edge. During the backing process, the driver usually needs to judge the stopping point by observing the rearview mirror and feeling the vehicle’s condition. To avoid judgment errors by the driver, which may cause the rock removal vehicles to roll off from the edge of the dump platform, the safety regulations of the dump require that a safety retaining wall be built at the edge of the dump platform to protect the rock removal vehicles unloading operation. This is to reduce the number of safety incidents in dump operations. However, factors such as precipitation infiltration, rock removal vehicle tires, and rolled rock impacts can damage the safety retaining wall locally, compromising its ability to prevent rock removal vehicles from rolling down and seriously threatening their safety. Thus, regular checks are necessary to ensure that all areas of the safety retaining wall parameters meet safety standards. Consequently, studies of dump safety should not only include deformation monitoring and landslide early warning analysis for the entire dump but also a health assessment of the safety retaining wall in the unloading area, for which there is currently insufficient research.

Currently, traditional methods are still used to inspect and evaluate safety retaining walls in open pits. These methods rely on the subjective experience of inspectors to determine dangerous areas of the safety retaining wall and acquire characteristic parameters using total stations, RTK, and other measurement tools, which is a time-consuming and labor-intensive process that poses certain risks to worker safety. As such, the need to achieve intelligent safety inspection of the safety retaining wall has become essential and is an integral part of creating an Intelligent Mine. To address this need, this study proposed an approach to assess the health condition of the safety retaining wall based on UAV [11] point-cloud data [12]. This approach achieves unmanned and rapid inspection of the safety retaining wall and capitalizes on the high density and accuracy of UAV point-cloud data to accurately reflect the 3D terrain information [13] of a dump. The collection process is also highly automated, convenient, and safe, which makes it feasible to implement a safety retaining wall extraction and safety assessment method based on UAV point-cloud data.

The accuracy of modeling and evaluating the safety retaining wall relies on the accurate extraction of various types of target point clouds. Non-ground point-cloud extraction is currently performed using filtering methods such as Progressive Morphological 1D/2D (PM1D/2D) [14,15], ATIN [16,17,18], and MLS [19] algorithms. However, the safety standard for the dump requires the safety retaining wall to have a height not less than 2/5 of the rock removal vehicle’s tire diameter, with top and bottom widths not less than 1/4 and 4/3 of the tire diameter, respectively. These dimensions are much smaller than the overall size of the dump, with relatively indistinct characteristics. As such, these algorithms are likely to misclassify the safety retaining wall point-cloud target as a ground point cloud. To address this issue, this study proposed the use of an elevation gradient filtering algorithm to extract the point cloud of the dump platform and slope. Using these as a basis, the point cloud of the unloading rock boundary can then be extracted and used to extract the point-cloud of the safety retaining wall.

The extraction of the point-cloud boundary of the dump’s unloading rock boundary depends on algorithms that can accurately identify the point-cloud boundary. Current algorithms [20] for boundary extraction include those based on geometric features of scattered point-cloud, such as K-NN [21] and alpha-shape [22] algorithms, as well as those based on triangular networks, including Delaunay triangular network boundary extraction [23]. While these methods can achieve better extraction of point-cloud boundary, the extracted data can often be still disordered, leading to complicated and less efficient subsequent processing. To address these issues, this study proposed the ordered crisscrossed scanning algorithm for extracting the point-cloud boundary of the dump platform. This algorithm enables automatic ordering of the point-cloud boundary, laying the foundation for subsequent extraction of the safety retaining wall point cloud.

The main contributions of this study can be summarised as follows:

(a) To solve the issue of incorrect extraction and omission during direct extraction of the safety retaining wall using existing filtering methods, a series of algorithms, namely the elevation gradient filtering algorithm and the ordered crisscrossed scanning algorithm, are proposed to achieve an indirect extraction of the safety retaining wall. The proposed methodology involves the following steps: First, the elevation gradient filtering algorithm is used to extract the platform and slope point clouds. Then, the ordered crisscrossed scanning algorithm is employed to extract the ordered unloading rock boundary feature point cloud. Subsequently, the cubic Bézier curve algorithm is used to interpolate the unloading rock boundary feature point cloud, and ultimately, the safety retaining wall point cloud is extracted by applying a range constraint based on the unloading rock boundary point cloud.

(b) In order to evaluate the health of the safety retaining wall, this study proposes the use of a “sampling” approach, where each area of the wall is profiled at regular intervals and the geometric parameters of each profile are calculated. These values are then compared to standard parameters for the safety retaining wall to evaluate the wall’s overall health. The specific methodology is as follows: First, the Alpha-shape surface reconstruction algorithm is used to construct a Mesh model [24] of the safety retaining wall. Next, at equal intervals on the ordered unloading rock point-cloud boundary, the profile location point-cloud is inserted. This point cloud is then used to profile the safety retaining wall Mesh model, extracting the height, top width, and bottom width characteristics of each profile. Finally, the safety level of each profile is classified by comparing the extracted parameters to the standard parameters. The safety level of the profile represents the area where the safety retaining wall is located, achieving health assessment results for each area of the safety retaining wall.

**Figure 1:**
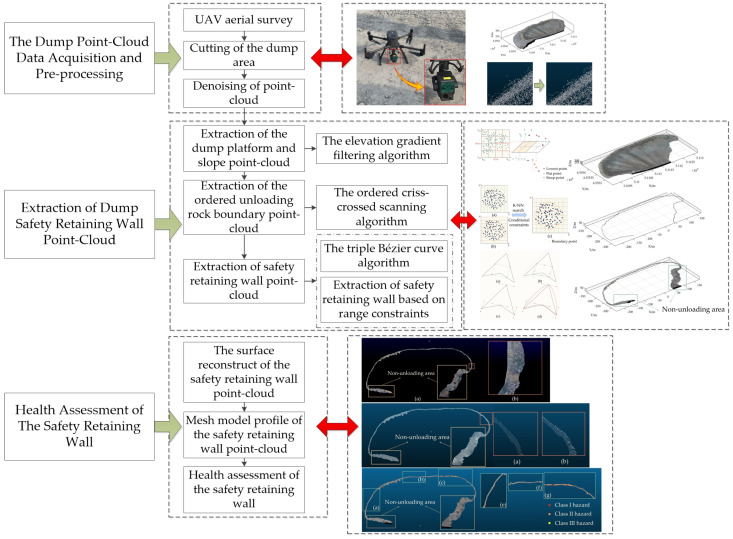
The flow chart of the safety retaining wall’s health assessment. The left side of the figure shows the three main parts of the thesis. In the middle are the specific processes for the three parts. On the right side is a schematic diagram of the algorithm for each process as well as a graph of the results.

The overall flow chart for this study is shown in Figure 1.

## 2. Materials and Methods

### 2.1. The Dump Point-Cloud Data Acquisition

#### 2.1.1. UVA Platform

**Figure 2:**
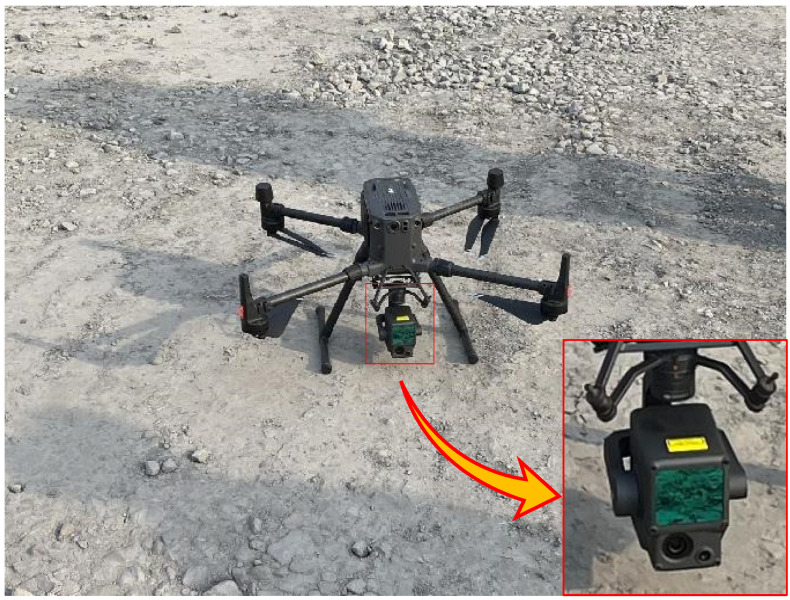
DJI MATRICE M300 RTK UAV.

The aerial survey equipment mainly comprises the DJI Matrice M300 RTK UAV and its mounted DJI Zenmus L1 LIDAR as shown in Figure 2, with the main technical parameters shown in Table 1 and Table 2. The main parameters of the Graphics Workstation used in the data processing are as follows: processor: AMD Ryzen 7 5800H CPU @ 3.2 GHz; graphics card: NVIDIAGeForceRTX3060; operating system: Windows 11 HomeBasic 64-bit; memory: 64 G. The software used to visualize the point-cloud data in this study is cloud compare. The softwares used to produce the figures were cloud compare, Matlab, WPS, Visio, and Arcgis. The programming language used for all algorithms in this study is Python, the compiler is Pycharm, and the point-cloud library is open3D.

#### 2.1.2. UVA Aerial Survey

**Figure 3:**
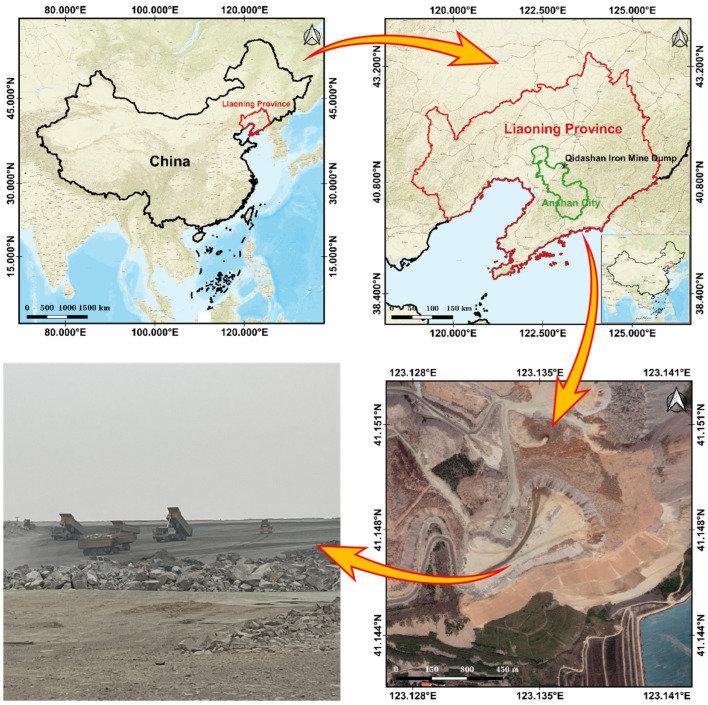
Survey area of the Qidashan Iron Mine Dump.

This study focused on the Qidashan Iron Mine Dump in Anshan City, Liaoning Province, China (as depicted in Figure 3) as the study area. The geographic coordinates of the dump’s center are 123°08′29.076″ E and 41°09′2.484″ N. The size of the dump is approximately 285 m in the north–south direction and 489.344 m in the east–west direction. The aerial survey data was collected on 17 February 2023, and the UAV was flown at an altitude of 100 m above the ground, at a speed of 6 m/s on average, with a duration of 13 min. The point cloud acquisition featured a number of echoes of 3 and had a laser bypass overlap of 60%.

### 2.2. Extraction of Dump Safety Retaining Wall Point-Cloud

The safety retaining wall features at the dump are relatively inconspicuous in comparison to the overall dump. This makes it challenging to extract the safety retaining wall directly using its features, as there is a risk of erroneously extracting rock blocks with similar features instead of the safety retaining wall. Additionally, the features of an unqualified safety retaining wall are even less conspicuous, which can lead to omitting the detection of certain safety retaining walls. To overcome these challenges, this study puts forward the concept of “indirect” extraction of the safety retaining wall. Firstly, the elevation gradient filtering algorithm was used to extract the point-cloud data of the dump platform and slope. Then, the ordered crisscrossed scanning algorithm was used to extract the point-cloud data of the ordered unloading rock boundary of the dump. Lastly, the extraction algorithm, which is based on the range constraint, was used to extract the safety retaining wall. This approach helps to provide a foundation for subsequent health assessments of the safety retaining wall.

#### 2.2.1. Extraction of the Dump Platform and Slope

The topography of the dump platform area is indeed flat, while the elevation gradients between the points of the safety retaining wall point-cloud vary widely due to its approximate trapezoidal or triangular shape. To extract the point cloud of the dump platform and slope, the elevation gradient filtering algorithm was employed. The steps involved in this process are as follows:

Use the elevation gradient filtering to extract the point-cloud of the dump platform and slope: Firstly, the point-cloud data are divided into virtual regular grids in the XOY plane along the X and Y axes respectively according to the set edge length l. The maximum values (${x_{max},y}_{max}$) and the minimum values (${x_{min},y}_{min}$) of the point-cloud data in the X and Y axes are used as the boundary of the regular grid division, and the regular grid is divided into rows and columns (*row col*). The specific formula is as follows:

(1) $$\begin{array}{l} row=\left[\frac{y_{\max}-y_{\min}}{l}\right]\\ col=\left[\frac{x_{\max}-x_{\min}}{l}\right] \end{array}$$

Each point in the point cloud is given a corresponding grid index, which is calculated as follows:

(2) $$\begin{array}{l} rowID=\left[\frac{y_{i}-y_{\min}}{l}\right]\\ colID=\left[\frac{x_{i}-x_{\min}}{l}\right] \end{array}$$

where (${x_{i},y}_{i}$) is the plane coordinate of any point; where (*rowID*, *colID*) is the number of the grid where the point is located.

The point with the smallest z-coordinate in each grid is determined, and the other points in the grid are calculated with that point for relative elevation and elevation gradient respectively. The specific formula is as follows:

(3) $$\begin{array}{l} \Delta z_{i}=z_{i}-z_{G\min}\\ \mathrm{gradient}=\frac{\Delta z_{i}}{\sqrt{{\left(x_{i}-x_{G_{\min}}\right)}^{2}+{\left(y_{i}-y_{G_{\min}}\right)}^{2}}} \end{array}$$

where $G_{min}$ is the minimum point in the *z*-axis direction in each grid; where (${x_{G_{min}},y}_{G_{min}},z_{G_{min}}$) is the coordinate of the minimum point in the *z*-axis direction in each grid.

**Figure 4:**
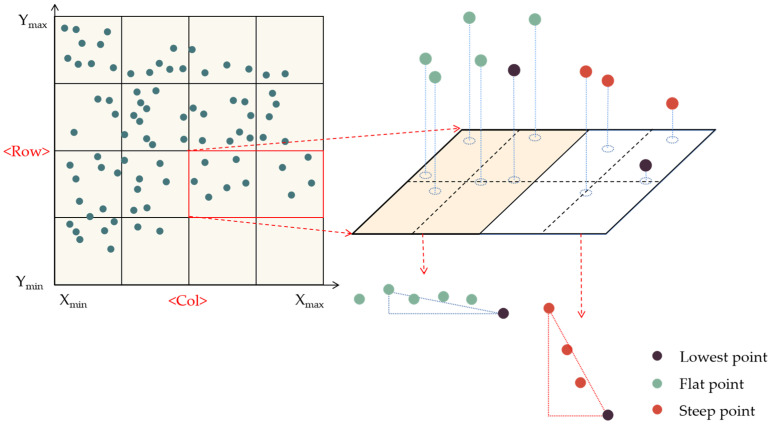
The elevation gradient filtering algorithm.

According to the set elevation threshold (*H*) and elevation gradient threshold (*G*), points less than the elevation threshold (*H*) and the elevation gradient threshold (*G*) will be extracted to achieve flat area point-cloud extraction. Points greater than the elevation threshold (*H*) and the elevation gradient threshold (*G*) are extracted to achieve point-cloud extraction in the steep area. The diagram of the elevation gradient filtering algorithm is shown in Figure 4.

#### 2.2.2. Extraction of the Ordered Unloading Rock Point-Cloud Boundary

Indirect extraction of the safety retaining wall crucially involves extracting the ordered unloading rock boundary point cloud. The process starts by extracting the boundary point-cloud of the dump platform from the previously extracted point-cloud data. Subsequently, this study constrains the platform boundary point-cloud using the slope point cloud to extract the ordered unloading rock boundary point-cloud.

This study proposes the use of an ordered crisscrossed scanning algorithm to extract the boundary point cloud of the dump platform. The algorithm first divides the point cloud into equal parts along the *x*-axis, and each part is enclosed by a minimum bounding box to locate the point with the largest Y-coordinate and the smallest Y-coordinate. Next, the point cloud is similarly divided into equal parts along the *y*-axis, with each part enclosed by a bounding box to obtain the point with the largest X-coordinate and the smallest X-coordinate. These identified points form the point-cloud boundary.

**Figure 5:**
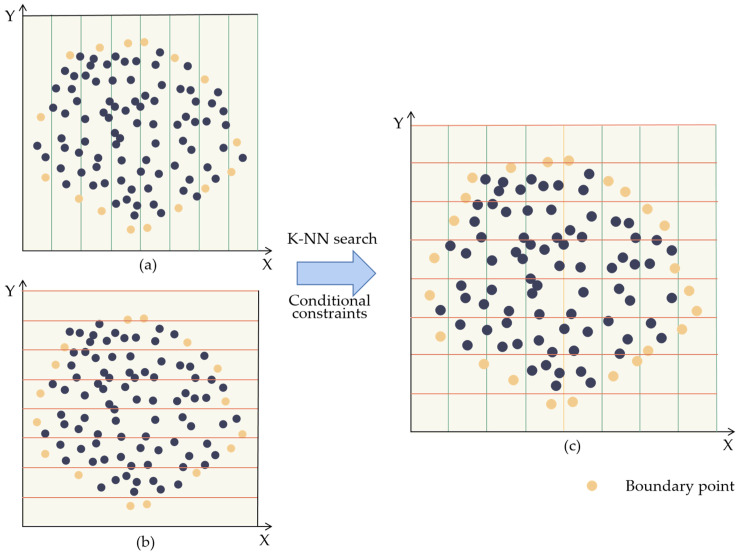
The ordered crisscrossed scanning algorithm. (**a**) Divide the point cloud into equal parts along the *x*-axis, and each part is enclosed by a minimum bounding box to locate the point with the largest y-coordinate and the smallest y-coordinate; (**b**) Divided into equal parts along the *y*-axis, with each part enclosed by a bounding box to obtain the point with the largest x-coordinate and the smallest x-coordinate; (**c**) Point cloud extraction at the boundary of the dump platform by the ordered crisscrossed scanning algorithm.

This is carried out as follows: The first step is to obtain the boundary information of the point-cloud data by creating the minimum enclosing box of the point cloud in the two-dimensional plane and calculating the extreme values (${x_{max},x}_{min}$) along the *X*-axis and the extreme values (${y_{max},y}_{min}$) along the *Y*-axis, respectively. According to the set parameter steps (*dx*), equal intervals are divided along the *X*-axis direction and *Y*-axis direction, respectively. Calculate the index of the grid corresponding to each point, and when in grids divided along the *X*-axis, calculate the maximum and minimum Y-value points in each grid, and arrange the minimum points in reverse order to obtain an ordered set of point-cloud (*A* = {*a_j_*}, (*j* = 1,2,…*n*)). Additionally, when in grids divided along the *y*-axis, calculate the maximum and minimum X-value points in each grid, and arrange the minimum points in reverse order to obtain an ordered set of point clouds (*B* = {*b_i_*}, (*i* = 1,2,…*n*)). Find the nearest-neighbor point (*a_n_*) of a point (*b_n_*) in point cloud *B* in point cloud *A* by the K-NN search algorithm. Then, calculate the distance (*d_n_*_−1_, *d_n_*_+1_) between point (*a_n_*) and point (*a_n_*_−1_), point (*a_n_*_+1_) respectively, and calculate the distance (*l_n_*_−1_, *l_n_*_+1_) between point (*b_n_*) and point (*a_n_*_−1_), point (*a_n_*_+1_). If *l_n_*_−1_ < *d_n_*_−1_ and *l_n_*_+1_ > *d_n_*_+1_, it is known that point (*b_n_*) lies between point (*a_n_*) and point (*a_n_*_−1_). If *l_n_*_−1_ > *d_n_*_−1_ and *l_n_*_+1_ < *d_n_*_+1_, it is known that point (*b_n_*) lies between point (*a_n_*) and point (*a_n_*_+1_). Iterate through point-cloud *B* and insert the eligible points into point-cloud *A* to achieve an ordered merging of boundary points. The nearest-neighbor search for the ordered point-cloud boundary is then carried out by traversing the slope point cloud, and the extraction of the ordered unloading rock point-cloud boundary is achieved within the constraints of the slope point-cloud. A diagram of the ordered crisscrossed scanning algorithm is shown in Figure 5.

#### 2.2.3. Extraction of the Safety Retaining Wall of the Dump Point-Cloud

To extract the point-cloud data of the safety retaining wall, it is necessary to remove the point-cloud datasets corresponding to the dump platform and the slope from the dump point cloud. This yields the desired safety retaining wall point cloud. As these two point-cloud datasets are situated on either side of the ordered dump platform point-cloud boundary, these point-cloud boundary data can be converted into a shape-line data to effectively segment the dump platform point-cloud from the non-dump platform point cloud. The ordered unloading rock point-cloud boundary is then interpolated and fitted. The non-dump platform point cloud is subsequently searched using a radius search algorithm, and then an elevation constraint, based on the ordered dump platform point-cloud boundary, is applied to achieve the desired extraction of the safety retaining wall point cloud.

(1) The point cloud from the ordered unloading rock boundary is interpolated and fitted. Previous research has shown that in practical engineering applications, the use of the Bézier method has proven to be more effective than the B-sample method. The triple Bézier curve is also found to handle inflection points better [25]. Therefore, this study proposes the use of a cubic Bézier curve interpolation algorithm to fit the point cloud obtained from the ordered unloading rock boundary [26]. The mathematical definition of a Bézier curve is shown below.

With *n* + 1 point vectors (${\left\{P_{i}\right\}}_{i=0}^{n}$), the curve corresponding to them is called an nth Bézier curve as follows.

(4) $$P\left(t\right)=\sum_{j=0}^{n}B_{j,n}\left(t\right)P_{j},0\leq t\leq 1$$

where *P*_0_, *P*_1_, … *P_n_* are called control polygons or feature polygons. *P_j_* (*j* = 0,1,…,*n*) are called control vertices, and *B_j,n_*(*t*) is the *n*th Bernstein basis function.

(5) $$B_{j,n}\left(t\right)=C_{n}^{i}t^{i}{\left(1-t\right)}^{n-i}$$

When *n* = 3, the cubic Bernstein basis function is obtained from Equation (5) as follows.

(6) $$\left\{\begin{array}{c} B_{0,3}\left(t\right)=C_{3}^{0}t^{0}{\left(1-t\right)}^{3}={\left(1-t\right)}^{3}\\ B_{1,3}\left(t\right)=C_{3}^{1}t^{1}{\left(1-t\right)}^{2}=3t{\left(1-t\right)}^{2}\\ B_{2,3}\left(t\right)=C_{3}^{2}t^{2}(1-t)=3t^{2}(1-t)\\ B_{3,3}\left(t\right)=C_{3}^{3}t^{3}{\left(1-t\right)}^{0}=t^{3} \end{array}\right.$$

Thus, the cubic Bézier curve can be expressed from Equation (4) as follows.

(7) $$\begin{array}{ll} P\left(t\right) & =\sum_{j=0}^{n}B_{j,3}\left(t\right)P_{j}\\ & =\left[{\left(1-t\right)}^{3}\;3t{\left(1-t\right)}^{2}\;3t^{2}(1-t)\;t^{3}\right]\left[\begin{array}{c} P_{0}\\ P_{1}\\ P_{2}\\ P_{3} \end{array}\right],0\leq t\leq 1 \end{array}$$

The above-shown equation can be rewritten in the form of a matrix product as follows.

(8) $$P\left(t\right)=\left[\begin{array}{cccc} 1 & t & t^{2} & t^{3} \end{array}\right]\left[\begin{array}{cccc} 1 & 0 & 0 & 0\\ -3 & 3 & 0 & 0\\ 3 & -6 & 3 & 0\\ -1 & 3 & -3 & 1 \end{array}\right]\left[\begin{array}{c} P_{0}\\ P_{1}\\ P_{2}\\ P_{3} \end{array}\right],0\leq t\leq 1$$

**Figure 6:**
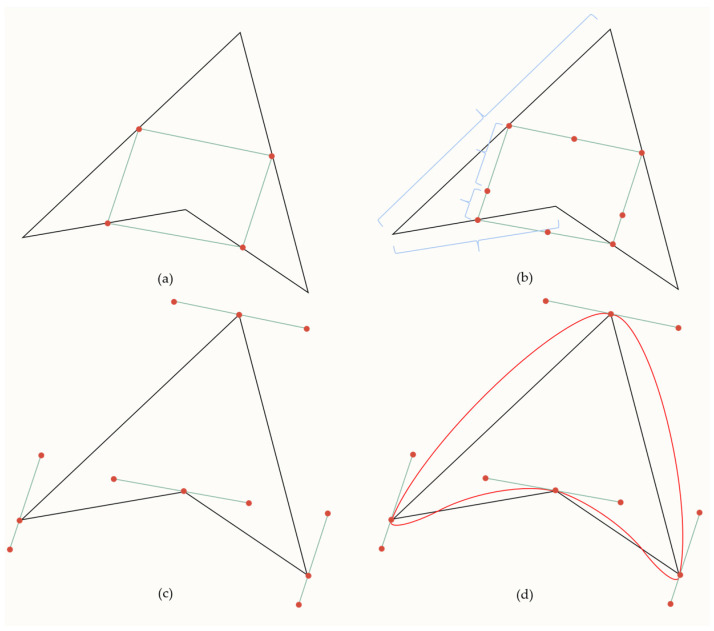
The triple Bézier curve algorithm. (**a**–**d**) A cubic Bézier curve implementation process. (**a**): The black line connects the adjacent original data points to calculate the midpoint of each line segment, and the green line connects the adjacent midpoints. (**b**): The green line joins the adjacent midpoints and marks the split point by dividing the green line between its midpoints according to the ratio of the lengths of the two adjacent black lines. (**c**): The green line is panned so that the split point coincides with the relative original data point, thus obtaining the control points of the cubic Bessel curve.

As can be seen above, a cubic Bézier curve can be constructed given a vector of four control vertices (*P*_0_, *P*_1_, *P*_2_, *P*_3_) of the characteristic polygon and using either Equation (7) or Equation (8). A diagram of the triple Bézier curve algorithm is shown in Figure 6.

(2) The proposed safety retaining wall extraction algorithm utilizes a range constraint approach to extract the safety retaining wall. The range constraint is mainly comprised of two parts. Firstly, the algorithm converts the point-cloud data of the dump platform boundary into shape-line data and segments the dump point-cloud into dump platform point-cloud and dump non-platform point-cloud by determining whether the points fall within or outside the boundary shape-line data. The non-dump platform point-cloud is then radius searched using the dump point-cloud boundary as the search center point, whereby points with elevations above that of the center point are identified as the safe retaining wall point-cloud. This approach enables the accurate extraction of safe retaining wall point-cloud data from the original dump point-cloud data.

### 2.3. Health Assessment of the Safety Retaining Wall

Due to the varying height and width of each area of the safety retaining wall, and the lack of significant patterns between them, it is not possible to represent the characteristics of each area by those of the overall safety retaining wall. To address this challenge, we propose a novel approach based on “sampling”. Specifically, we used the Alpha-shape algorithm to perform surface reconstruction of the safety retaining wall, and then divide the resulting Mesh model into equal intervals to extract sample profiles of each area. By analyzing the extracted profile characteristics and comparing them with normative parameters of the safety retaining wall in the dump, we can enable the health assessment of different areas of the retaining wall. The health class of each sample profile corresponds to the health class of its corresponding area in the safety retaining wall.

#### 2.3.1. The Surface Reconstruct of the Safety Retaining Wall Point-Cloud

In order to obtain the profile parameters of the safety retaining wall to evaluate its safety condition, a surface reconstruction method based on Alpha-shape was introduced to reconstruct the surface of the safety retaining wall from the scattered and disordered set of points. The specific algorithm steps are as follows:

(1) Select any point (*q*_1_) from the safety retaining wall point-cloud (*Q* = {*q_j_*}, (*j* = 1,2,…*n*)). A new point-cloud (*Q*_1_) is formed by selecting any point from the security retaining wall point-cloud that is <2α away from it. Take any set of points (*q*_2_, *q*_3_) from the point-cloud (*Q*_1_). Find the centers (*o*, *o*′) of the balls passing through points (*q*_2_, *q*_3_, *q*_1_) and having a radius (α).

(2) Traverse the point-cloud (*Q*_1_) and find sets (*l*, *l*′) of distances from the other points to the centers (*o*, *o*′) of the balls in turn. If the distances from sets (*l*, *l*′) are all >α, then the points (q_2_, q_3_, q_1_) can be judged to be edge contour points, connecting the three points to form a boundary triangle. If not, then it is not an edge contour point, stop traversing and perform step (3).

(3) Select the next set of points in the point-cloud (*Q*_1_) and follow steps (1) and (2) until all points in the point-cloud (*Q*_1_) are judged and the set (Δ) of facets is output. The exposed triangular face pieces in the set form a local convex package (*δS*α(*P*)), calculated as shown in Equation (9):

(9) $$\delta S_{\alpha }\left(Q\right)=\left\{\Delta |Q_{1}\subset Q\cap |Q_{1}|\leq 2\alpha \right\}$$

(4) Select the next point in the point cloud (*Q*) to be judged according to steps (1) to (3) until all points in the point cloud (*Q*) are judged and multiple local convex packages (*δS*α(*P*)) reconstruct the surface.

#### 2.3.2. Mesh Model Profile of the Safety Retaining Wall Point-Cloud

In order to accurately obtain the characteristic parameters of each area of the safety retaining wall, we introduced the idea of sampling by performing equally spaced profiles on the point cloud of the safety retaining wall after surface reconstruction. The profile characteristic parameters were then extracted to represent the characteristic parameters of the entire safety retaining wall. To locate the profile location point on the extracted ordered unloading rock boundary, we interpolated the profile location point. The tangent vector of the profile location point was then calculated. The corresponding plane to the point was inverted with the tangent vector serving as the normal vector. Finally, the plane intersected its Mesh model within a certain range to obtain the profile point and determine the profile at that location, as follows:

(1) First determine the position of the profile location point. The total (*D*) Euclidean distance between adjacent points is calculated in the ordered unloading rock point-cloud boundary. Interpolation of *D*/*d* profile position points is performed according to the set interval distance (*d*). The following Equation (10) allows one to determine that the profile location point (*o*) is located between the points (*o_i_*_+1_, *o_i_*_+2_), and then, according to Formula (11), we can calculate the coordinates of the profile location point.

(10) $$\sum_{i=1}^{n-1}d_{i}<m\times d\leq \sum_{i=1}^{n-1}d_{i+1}$$

In the formula, m=1,2,⋯D/d.

(11) $$\left(\begin{array}{ccc} x_{o} & y_{o} & z_{o} \end{array}\right)=\left\{\begin{array}{c} x_{o}=\left(x_{o_{i+2}}-x_{o_{i+1}}\right)t+x_{o_{i+1}}\\ y_{o}=\left(y_{o_{i+2}}-y_{o_{i+1}}\right)t+y_{o_{i+1}}\\ z_{o}=\left(z_{o_{i+2}}-z_{o_{i+1}}\right)t+z_{o_{i+1}} \end{array}\right.⇐\frac{x_{o}-x_{o_{i+1}}}{x_{o_{i+2}}-x_{o_{i+1}}}=\frac{y_{o}-y_{o_{i+1}}}{y_{o_{i+2}}-y_{o_{i+1}}}=\frac{z_{o}-z_{o_{i+1}}}{z_{o_{i+2}}-z_{o_{i+1}}}=t$$

(2) The tangent vectors are calculated for the profile location point on the ordered unloading rock boundary. The nearest neighbor search algorithm is used to find the nearest neighbor of the profile location point and the two points are calculated as a vector with the following equation:

(12) $$LineVector=(x_{1}-x_{2},y_{1}-y_{2},z_{1}-z_{2})=(m,n,p)$$

(3) The tangent vector is used as a normal vector to invert the plane equation and calculate the profile points. The basic definition of a plane equation: the equation corresponding to all points in three dimensions that are in the same plane. Any plane can be represented by a ternary equation. According to the basic formula of the plane equation (Ax+By+Cz+D=0), as well as the coordinates (x,y, *z*) and normal vectors (m,n,p) of the dissection points already obtained, the coefficients of the plane equation can be obtained according to the point normal of the plane equation, with the following formula:

(13) $$\begin{array}{l} \;n\left(x-x_{o}\right)+m\left(y-y_{o}\right)+p\left(z-z_{o}\right)=0\\ \Rightarrow nx+my+pz+\left(-nx_{o}-my_{o}-pz_{o}\right)=0 \end{array}$$

The plane equation coefficients are A=n, B=m, C=p, and D=(−nx−ny−pz).

The Mesh model of the safety retaining wall is stored as three vertex indexes for each triangular face piece so that each edge of each triangular face piece is represented by the vertex corresponding to the edge. The set of edges ($E=\left\{e_{j}\right\},(j=1,2,\cdots n)$) of the Mesh model of the security retaining wall is traversed and judged according to the equation, which is satisfied as the edge $\left(e(q_{1}q_{2})\right)$ that intersects the profile.

(14) $$\left\{\begin{array}{c} Ax_{1}+By_{1}+Cz_{1}+D<0\\ Ax_{2}+By_{2}+Cz_{2}+D>0 \end{array}\right.$$

Knowing the equation coefficients A,B,C, and *D* and the side $\left(e(q_{1}q_{2})\right)$ being dissected, the intersection (q) of the line with the face can be calculated using the following formula.

(15) $$\left\{\begin{array}{c} Ax_{q}+By_{q}+Cz_{q}+D=0\\ \frac{x_{q}-x_{q_{1}}}{x_{q_{2}}-x_{q_{1}}}=\frac{y_{q}-y_{q_{1}}}{y_{q_{2}}-y_{q_{1}}}=\frac{z_{q}-z_{q_{1}}}{z_{q_{2}}-z_{q_{1}}}=t \end{array}\right.$$

#### 2.3.3. Health Assessment of the Safety Retaining Wall

This study proposes a method for assessing the safety and health of retaining walls by analyzing the height, top width, and bottom width of equally spaced profiles, which are then compared with the standard parameters. A grade assessment is introduced to classify the health of retaining walls into four categories, namely safe, and dangerous (with three levels of severity). A retaining wall is considered safe if all three parameters meet the criteria, while it is deemed dangerous if one or more parameters do not meet the standards. A Class 1 hazard is assigned if one parameter fails, Class 2 if two parameters fail, and Class 3 if all three parameters fail.

After the safety retaining wall profile has been assessed for the finished safety health rating, it is fed back to the safety retaining wall point-cloud data for a regional health rating assessment. The specific steps are as follows: The profile’s location points and the plane equation coefficients of the profile are known. The health assessment of the safety retaining wall is achieved by aligning the points located at a distance of less than or equal to d/2 in the safety retaining wall point cloud on both sides of the profile with the health assessment level of the profile.

## 3. Results

### 3.1. UAV Point-Cloud Data

**Figure 7:**
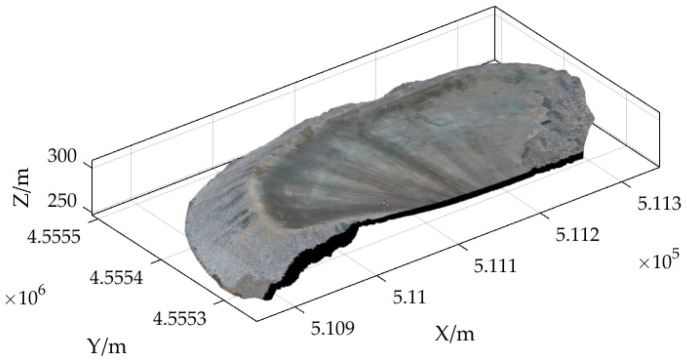
Qidashan Iron Mine Dump point-cloud data.

The UVA point-cloud data were cropped and only the point-cloud data in the dump field area were retained to form the dump point-cloud model shown in Figure 7. The relevant parameters for these point-cloud data are shown in Table 3.

### 3.2. Extraction of Dump Safety Retaining Wall Point-Cloud

#### 3.2.1. Extraction of the Dump Platform and Slope

**Figure 8:**
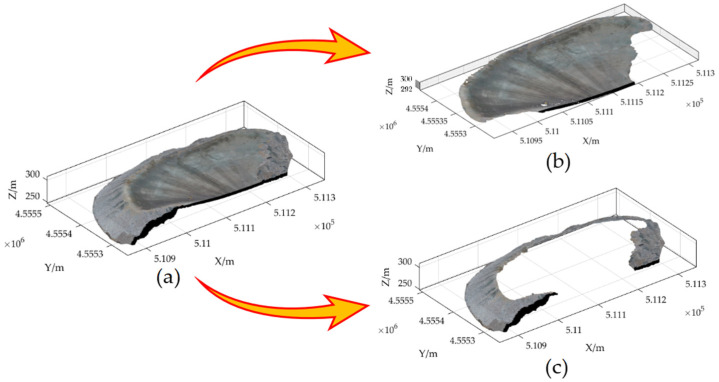
The elevation gradient filtering algorithm is used to extract the point cloud of the dump platform and slope. (**a**) Dump point-cloud data; (**b**) the point-cloud of the dump platform; (**c**) the point-cloud of the dump slope.

To extract the point cloud of the dump platform and slope, an elevation gradient filtering algorithm was used. The algorithm began by creating a regular grid of point-cloud data in the XOY plane for the entire dump, with an edge length of 1 m for each grid. The algorithm then identified the lowest point in each grid and calculated the gradient of elevation and the difference in elevation between the lowest point and the other points. As the height of the standard safety retaining wall model is 0.576 m, a height difference threshold of 0.3 m was set in order to avoid the point cloud of the substandard safety retaining wall being mistaken for the ground point cloud. As the platform of the dump is required to be flat, the elevation gradient between the highest and lowest point of the point cloud within a unit square meter does not exceed 0.15, and to increase the robustness of the algorithm, an elevation gradient threshold of 0.2 was set. When both the elevation difference and the elevation gradient were less than the thresholds, the point-cloud data of the dump platform was extracted. Likewise, when both the elevation difference and the elevation gradient were higher than the thresholds, the point-cloud data of the dump slope were extracted. The results are shown in Figure 8.

#### 3.2.2. Extraction of the Ordered Unloading Rock Point-cloud Boundary

To extract the point-cloud of the ordered unloading rock boundary between the dump platform and the safety retaining wall, the ordered criss-crossed scanning algorithm was used to extract the boundary of the dump platform. The step size for zoning along the X and Y axes was set to 1 m, based on the size of the abrupt changes in features at the ordered unloading rock boundary. The special features of this algorithm not only allow for the extraction of point-cloud boundary but also make them ordered. The nearest-neighbor search was carried out on the dump platform point-cloud boundary by traversing the slope point cloud. In this way, the extraction of the point cloud of the ordered unloading rock boundary was achieved.

**Figure 9:**
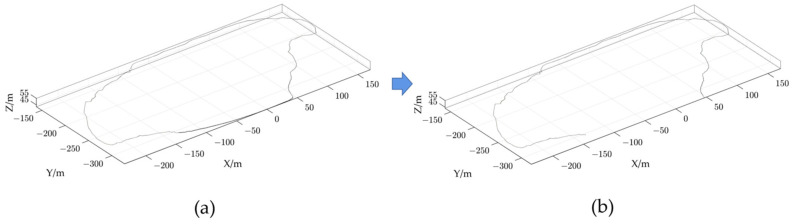
The ordered crisscrossed scanning algorithm was used to extract the boundary of the dump platform. (**a**) The point-cloud boundary of the dump platform; (**b**) The ordered unloading rock point-cloud boundary.

To reduce the memory consumption of big clouds, cloudcompare stores their points coordinates on 32 bits (single-precision floating-point format). This “big coordinates” issue typically arises when an object a few hundred meters wide is expressed in a global geographic coordinate system. Importing such coordinates in 32 bits format will result in a precision of several centimeters or worse. As the absolute position of clouds is generally not used during a comparison process (and most of the other processings) the best solution to this “big coordinates” issue is to temporarily shift the data to a local coordinate system. The inverse shift will be applied to the data at export time so that no information is lost. The results are shown in Figure 9.

#### 3.2.3. Extraction of the Safety Retaining Wall of the Dump Point-Cloud

**Figure 10:**
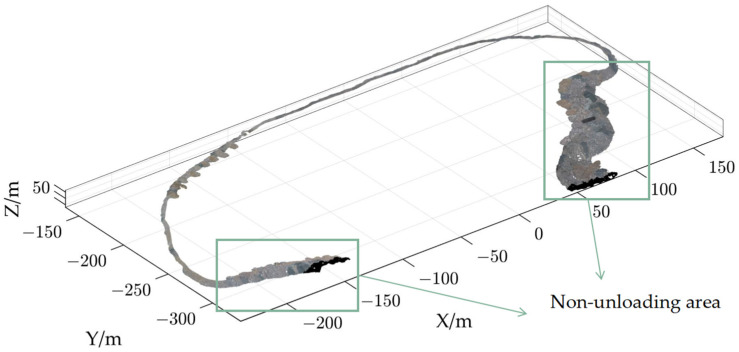
The safety retaining wall of the dump point-cloud.

The ordered unloading rock boundary was smoothly fitted using a cubic Bézier curve interpolation algorithm. A safety retaining wall point cloud was then extracted based on a range constraint at the ordered unloading rock boundary. The ordered unloading rock point-cloud boundary was converted to shape-line data. The dump point cloud was split into two point clouds: a platform point cloud and a non-platform point cloud using the ordered unloading rock point-cloud boundary. The split was determined by checking whether the dump point cloud is inside or outside the boundary line. To extract the safety retaining wall, a 30 m radius search of the non-dump platform point cloud was carried out by traversing the ordered unloading rock point-cloud boundary as the centroid of the radius search. An elevation constraint was then applied to the non-dump platform point cloud based on the centroid elevation of the radius search. The 30 m radius search was set due to a maximum width of 30 m in the non-unloading area of the safety retaining wall. The results of this method are presented in Figure 10.

### 3.3. Health Assessment of the Safety Retaining Wall

#### 3.3.1. The Surface Reconstruct of the Safety Retaining Wall Point-Cloud

**Figure 11:**
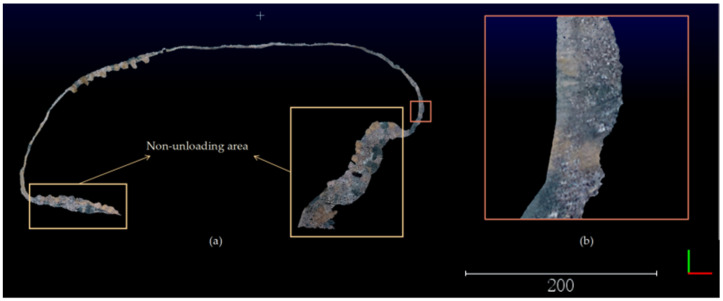
The surface reconstruction algorithm based on Alpha-shape is used to reconstruct the surface of the safety retaining wall. (**a**) The surface reconstruction of the safety retaining wall point-cloud; (**b**) The enlarged area of the partially reconstructed safety retaining wall on the surface is 10 times larger in side length compared to the original area. The bottom right corner of the diagram shows the axes, green for the *Y*-axis and red for the *X*-axis.

To obtain the profile features of the safety retaining wall, a surface reconstruction algorithm based on Alpha-shape was used to reconstruct the surface of the safety retaining wall. The parameter of this algorithm is the radius of the rolling ball, which is used as the radius of the rolling ball by calculating the average distance from each point to the 10 nearest-neighbor points using the kd-tree to achieve adaptivity. If too few k-nearest-neighbor points are selected, the average distance tends to be too large or too small for instability, resulting in missing features or holes in the Mesh model. If too many k-nearest-neighbor points are selected, it will increase the algorithm time, as shown in Figure 11.

#### 3.3.2. Mesh Model Profile of the Safety Retaining Wall Point-Cloud

**Figure 12:**
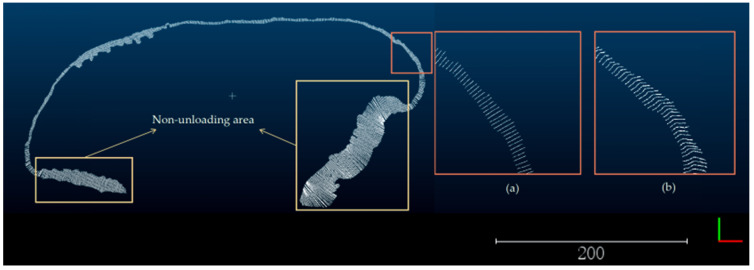
Profile of the safety retaining wall Mesh model with equal spacing. The edge lengths of the two enlarged areas in (**a**,**b**) are enlarged by a factor of 3.5 compared to the original area. (**a**) As can be seen in the top view of the profile point-cloud data, each of the profile point-cloud is on a straight line; (**b**) as can be seen from the side view, the point-cloud data of the profile can be representative of the safety retaining wall situation. The bottom right corner of the diagram shows the axes, green for the *Y*-axis and red for the *X*-axis.

To profile the safety retaining wall Mesh model with equal spacing, the first step is to determine the profile location and interpolate the profile location points based on the fitted dump. In this study, the equal spacing parameter is set to 1 m, and the regions of the safety retaining wall features 0.5 m on either side of the profile are represented by the profile characteristics, as shown in Figure 12.

#### 3.3.3. Health Assessment of the Safety Retaining Wall

**Figure 13:**
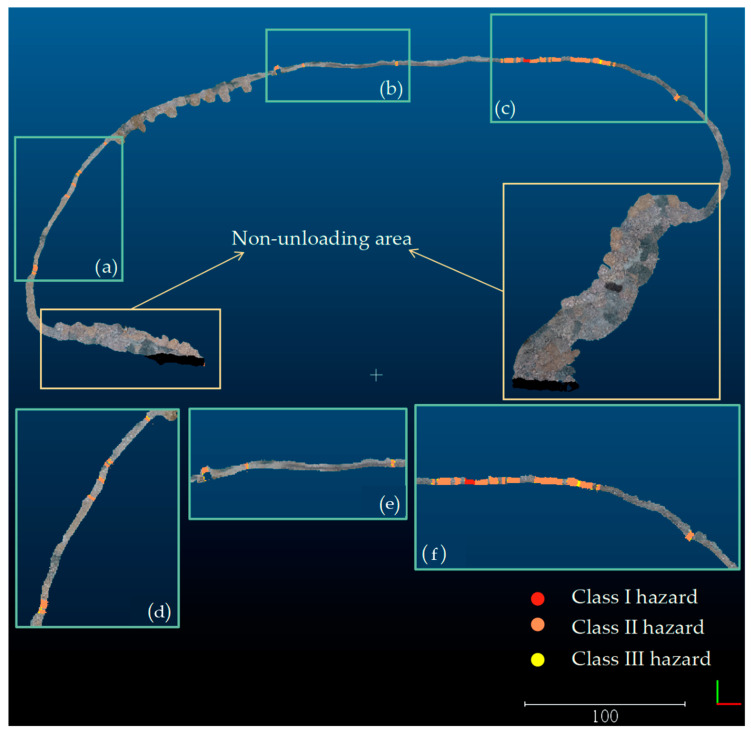
Health assessment of the safety retaining wall. The enlarged areas (**d**–**f**) correspond to the original areas (**a**–**c**), respectively, and the enlarged areas have 1.5 times larger side lengths compared to the original areas. The bottom right corner of the diagram shows the axes, green for the *Y*-axis and red for the *X*-axis. (**a**–**c**) The three safety retaining wall areas where hazards exist are represented in red for Class I hazards, orange for Class II hazards, and yellow for Class III hazards, with the original color of the retaining wall being qualified.

The assessment of the safety retaining wall is based on three critical profile parameters: height, top width, and bottom width. The safety standards for the dump require the safety retaining wall to be at least 2/5 of the tire diameter in height and no less than 1/4 and 4/3 of the tire diameter in top and bottom width, respectively. By researching the rock removal vehicles from this dump, it was identified that the LGMG MT96L mining dump truck has the largest outer diameter of 1439 mm tires for this model of vehicle. Consequently, the normative height for the safety retaining wall was 0.576 m, the normative top width was approximately 0.480 m, and the normative bottom width was 1.871 m. The individual profile characteristics are compared with the standards and graded accordingly. A retaining wall is considered safe if all three parameters meet the criteria, while it is deemed dangerous if one or more parameters do not meet the standards. If one parameter fails the criteria, it is a Class I hazard; if two parameters fail the criteria, it is a Class II hazard; and if all three parameters fail the criteria, it is a Class III hazard. An area of 0.5 m on either side of the profile is classified as having the same health condition based on the health of the profile derived from the profile in the safety retaining wall, as shown in Figure 13.

## 4. Discussion

### 4.1. Quantitative Evaluation of Extraction of Dump Safety Retaining Wall Point-Cloud

The safety retaining wall extraction algorithm based on the ordered unloading rock boundary for range constraint has been shown to produce better results for extracting safety retaining wall point clouds from the Qi Dashan Iron Mine Dump point cloud, as depicted in Figure 10. To evaluate the accuracy of the extraction results, this study employs the statistical error criterion proposed by the International Society for Photogrammetry and Remote Sensing (ISPRS) and the error rate of the safety retaining wall extraction results into three types: Type-I error, Type-II error, and total error. The Type-I and Type-II errors assess the method’s adaptability, while the total error reflects its feasibility, with a smaller total error indicating a more accurate extraction of the safety retaining wall point cloud. The equations for Type-I error, Type-II error, and total error are as follows.

(16) $$\alpha =\frac{b}{a+b}$$

(17) $$\beta =\frac{c}{c+d}$$

(18) $$\theta =\frac{b+c}{a+b+c+d}$$

In the above-shown equations, α represents the Type-I error, *β* represents the Type-II error, *θ* represents the total error, *a* denotes the number of points that are correctly classified as safe retaining wall points, *b* refers to the number of points that are misclassified as non-safe retaining wall points, *c* represents the number of points that are misclassified as safe retaining wall points while they are non-safe retaining wall points, and *d* stands for the number of points that are correctly labeled as non-safe retaining wall points.

The evaluation of the accuracy of the point-cloud extraction results for the safety retaining wall of the dump in this study, using the above-mentioned assessment method, is presented in Table 4.

Table 4 indicates that the point-cloud extraction method proposed in this study for the safety retaining wall of the dump has superior results. The average Type-I error, Type-II error, and total error of the safety retaining wall point-cloud extraction results were 0.22%, 0.47%, and 0.43%, respectively, indicating that all three types of error can be effectively controlled within a relatively small range.

### 4.2. Quantitative Evaluation of Health Assessment of the Safety Retaining Wall

The results of the health assessment of the safety retaining wall are presented in Figure 13, which displays the health level of different areas of the retaining wall. To evaluate the effectiveness of the health assessment quantitatively, the height, top width, and bottom width of selected areas of the safety retaining wall were measured using a steel ruler. For the safety retaining wall, the normative height was 0.576 m, the normative top width was approximately 0.480 m, and the normative bottom width was 1.871 m. The results of the quantitative evaluation of the health assessment effects of the safety retaining wall are presented in Table 5.

As depicted in Table 5, all three parameters (height, top width, and bottom width) measured in the corresponding model area had the centimeter-level error control. Among the 10 sampled areas, the top and bottom width parameters of the measured area 1 did not meet the standard, resulting in the corresponding model area belonging to the Class-II hazard area where two parameters failed to meet the standard. Additionally, the top width parameters of the measured areas 2 and 6 did not meet the standard, leading to the corresponding model area belonging to the Class-I hazard area where only one parameter failed to meet the standard. All other parameter values met the standard, with the corresponding model areas deemed safe. Thus, this safety wall health assessment method effectively reflects the health condition of each area of the safety wall in the field.

## 5. Conclusions

The traditional inspection process for the safety retaining wall in dumps is very expensive in terms of manpower, materials, and financial resources. Moreover, the inspection process can pose safety hazards. To address these concerns, this study proposed a novel approach. Firstly, point-cloud data of the dump platform and slope were extracted separately using elevation gradient filtering. Next, the ordered crisscross scanning algorithm was used to extract point-cloud data of the ordered unloading rock boundary features. The point-cloud data of the safety retaining wall were then extracted using a range constraint algorithm, and surface reconstruction was carried out to construct a mesh model. The safety retaining wall model was then isometrically divided to extract profile feature information, which is compared to the standard parameters of the safety retaining wall. Finally, a health assessment of the safety retaining wall was performed, facilitating rapid inspection of the wall. In conclusion, this study presents a novel approach to address the challenges of traditional safety retaining wall inspection, and the proposed method showed promising potential for efficient and effective safety retaining wall inspection. This study’s key contributions can be summarised as follows:

(a) This study presents a novel approach for the automatic extraction of safety retaining walls in dumps using a series of algorithms, including elevation gradient filtering and ordered crisscrossed scanning. The proposed method effectively overcomes the problems of false extraction and omission in traditional filtering algorithms. The ordered crisscrossed scanning algorithm extracts the unloading rock boundary more robustly than other boundary extraction algorithms, such as alpha-shape, and provides an ordered point cloud of the unloading rock boundary. Accurate extraction of the ordered unloading rock boundary is crucial for the range constraint method employed for extracting the safety retaining wall. Based on the quantitative evaluation of the safety retaining wall extraction, the Type-I error, Type-II error, and Total error were 0.22%, 0.47%, and 0.43%, respectively, demonstrating high accuracy and feasibility. The results show that the proposed algorithm is capable of accurately and automatically extracting the safety retaining wall in dumps and can provide technical support for subsequent health assessment of the safety retaining wall. Overall, this study provides a promising method for cost-effective and safer safety retaining wall inspections in dumps;

(b) This study proposed a novel assessment approach to obtain a safety retaining wall profile by reconstructing the surface from the safety retaining wall point cloud and performing profiling, overcoming the limitations of other methods, such as slicing and projecting, which generate profiles with significant errors. The quantitative evaluation results indicate that the height, top width, and bottom width parameters obtained from the field measurements for 10 sampled areas demonstrate centimeter-level accuracy and consistency with the corresponding positions in the point-cloud model. The safety retaining wall health assessment model matches the health assessment of the area in which the model is located, enabling accurate monitoring of the health of the safety retaining wall and the alerting of potential hazards, facilitating speedy inspections of the safety retaining wall. Overall, the proposed method has substantial potential for efficient and effective safety retaining wall monitoring.

There is a limitation of the presented method in this study. The study is based on orthorectified point-cloud data acquired by a drone-mounted LiDAR. If rock removal vehicles are performing soil unloading during data acquisition, the rear bucket of the rock removal vehicles may obstruct part of the safety retaining wall. When non-geological target point clouds, such as rock removal vehicles, are removed from the dump point-cloud data, the safety retaining wall point-cloud data will be incomplete. Therefore, in subsequent health assessments of the safety retaining wall, the top width and bottom width parameters of the incomplete section of the safety retaining wall may be underestimated, leading to a misjudgment that originally qualified walls are unqualified when compared to the standard.

## Figures and Tables

**Table 1:** Main technical parameters of the DJI Matrice M300 RTK UAV.

Aerial-Survey Equipment	Parameter	Value
UVA	Hovering accuracy (P-GNSS)	Vertical: ±0.1 m (visual positioning normal operation) ±0.5 m (GNSS normal operation) ±0.1 m (RTK positioning normal operation) Horizontal: ±0.3 m (visual positioning normal operation) ±1.5 m (GNSS normal operation) ±0.1 m (RTK positioning normal)
	RTK Position Accuracy	At RTK FIX: 1 cm + 1 ppm (horizontal) 1.5 cm + 1 ppm (vertical)
	Max. withstandable wind speed	15 m/s (12 m/s for take-off and landing phases)

**Table 2:** Main technical parameters of the DJI Zenmus L1 LIDAR.

Aerial-Survey Equipment	Parameter	Value
Lidar	Distance measuring accuracy (RMS 1σ)^2^	3 cm @ 100 m
	Max. number of echoes supported	3
	FOV	Repeated scans: 70.4° × 4.5°; Non-repetitive scans: 70.4° × 77.2°

**Table 3:** Parameters related to the point-cloud data of the dump.

Parameter	Value
Number of point-cloud	22,218,142
Average point distance (m)	0.049
Range (m × m × m)	486.993 × 258.232 × 58.316
Elevation (m)	250.467–308.783

**Table 4:** Error assessment of extraction results for safety retaining wall point sets.

*a*	*b*	*c*	*d*	*α*/%	*β*/%	*θ*/%
4,504,392	9873	105,994	17,607,756	0.22	0.60	0.52

**Table 5:** Error assessment of extraction results for safety retaining wall profile parameters.

Area	Measured			Model			Error			Level
	Height (m)	Top Width (m)	Bottom Width (m)	Height (m)	Top Width (m)	Bottom Width (m)	Height (m)	Top Width (m)	Bottom Width (m)	
1	0.700	0.358	1.781	0.707	0.313	1.770	0.007	0.045	0.011	Class II hazard
2	0.636	0.461	2.753	0.655	0.436	2.770	0.019	0.025	0.017	Class I hazard
3	1.009	0.749	2.523	1.024	0.730	2.493	0.015	0.019	0.03	qualified
4	0.901	0.787	2.912	0.889	0.795	2.884	0.012	0.008	0.028	qualified
5	0.726	0.756	2.088	0.710	0.660	2.086	0.016	0.096	0.002	qualified
6	0.755	0.327	2.028	0.753	0.284	2.035	0.002	0.043	0.007	Class I hazard
7	0.998	0.700	2.824	1.001	0.678	2.803	0.003	0.022	0.021	qualified
8	0.975	1.020	2.813	1.002	1.001	2.803	0.027	0.019	0.01	qualified
9	0.744	0.678	2.037	0.753	0.677	2.035	0.009	0.001	0.002	qualified
10	0.983	0.815	1.916	0.909	0.813	1.929	0.074	0.002	0.013	qualified

## Data Availability

The data presented in this study are available upon request from the corresponding author. The data are not publicly available due to privacy issues.
